# The Amyotrophic Lateral Sclerosis House Call Program: A Single‐Center Experience in the United States

**DOI:** 10.1155/nri/6629960

**Published:** 2026-02-27

**Authors:** Erica Scirocco, Jennifer Scalia, Benedetta Ugolini, Gabriella Casagrande, Doreen Ho, Jennifer L. Hagar, Kristen Kingsley, Ron Hoffman, Christopher Cooley, Merit E. Cudkowicz, Sabrina Paganoni, James D. Berry

**Affiliations:** ^1^ Harvard Medical School, Massachusetts General Hospital, Sean M. Healey and AMG Center for ALS & the Neurological Clinical Research Institute, Boston, Massachusetts, USA, harvard.edu; ^2^ Compassionate Care ALS, Falmouth, Massachusetts, USA; ^3^ Harvard Medical School, Spaulding Rehabilitation Hospital, Boston, Massachusetts, USA, spauldingrehab.org

**Keywords:** amyotrophic lateral sclerosis, healthcare access, house calls, multidisciplinary care, neuropalliative care

## Abstract

**Background:**

Accessing multidisciplinary care poses challenges for people living with amyotrophic lateral sclerosis (ALS) due to mobility issues. As ALS care rarely requires hospital‐based technology, most care is available through home visits. The Daniella Lipper ALS House Call Program (HCP) at Massachusetts General Hospital (MGH), launched in 2017 in collaboration with Compassionate Care ALS, has pioneered home‐based ALS care in Eastern Massachusetts.

**Methods:**

A retrospective chart review of ALS and primary lateral sclerosis (PLS) patients enrolled in the HCP at MGH was conducted. Data on demographics, visit details, and procedures performed during home visits were collected from electronic health records for patients seen from January 2024 to December 2024.

**Results:**

In 2024, the ALS HCP conducted 959 visits for 142 patients (average age: 68 years, range: 36–93; 47.9% female). Of these patients, 137 (96.5%) were diagnosed with ALS and 5 (3.5%) with PLS. Notably, 61 patients (43%) received care exclusively at home. Key interventions included 44 gastrostomy tube exchanges and 59 respiratory assessments, both of which significantly reduced hospital visits. The average distance traveled by the care team was 30.32 miles per visit.

**Conclusions:**

The Daniella Lipper ALS HCP at MGH brings ALS expertise into the patient’s home, minimizing travel burdens and ensuring continuity of care. The program illustrates the feasibility and impact of home‐based ALS care, suggesting potential for broader implementation across the nation. Development will focus on expanding services, such as tracheostomy changes in the homes, and on creating sustainable models for similar initiatives.

## 1. Introduction

Amyotrophic lateral sclerosis (ALS) is a progressive neurodegenerative disease with heterogeneous symptoms such as weakness, fatigue, spasticity, dysphagia, dysarthria, respiratory weakness, and, sometimes, behavioral and cognitive changes [1]. Multidisciplinary care is supported by international guidelines [2, 3] and offers multiple benefits, including both longer survival and a better quality of life [4–6], along with the timely introduction of palliative care [7]. Multidisciplinary clinics afford patients the opportunity to see several types of specialists during one visit. This facilitates treatment of several symptoms, exploration of research opportunities, and development of trusted relationships across specialties the patient may require in the future. However, as the disease progresses, people living with ALS experience physical disability, causing travel to a clinical center to become more burdensome. This, in turn, reduces access to multidisciplinary care and research, since these care centers are typically centralized at large academic centers [8, 9].

In the early 2010s, telemedicine programs, including the one at Massachusetts General Hospital (MGH) in Boston (US), began to supplement in‐clinic care for ALS patients [10]. These programs expanded substantially during the COVID‐19 pandemic and even became useful as a part of ALS research [11–14], easing the burden of travel and reducing patient costs [15]. However, telemedicine may not always be the best option to fully supplant in‐person evaluations, particularly in complex clinical situations that require patient examination or procedures [16].

House call visits, in which medical care is provided by physicians (MD/DO), nurse practitioners (NPs), or registered nurses (RNs) in the patient’s home, bear some resemblance to telemedicine in that they are also aimed at reducing patient burden and barriers to care, particularly for those with functional impairment from ALS. Beyond this, house calls permit full physical and neurological examinations, may more effectively involve family or other home care team members, and allow providers to assess the home environment and adjust care accordingly [17]. In ALS, house calls may reduce the need for hospitalization, particularly for pulmonary care and assessment, gastrostomy tube (G‐tube) exchange, access to research, and palliative care [17].

There is an emerging understanding of the role of house calls in improving satisfaction and potentially even survival of people living with ALS [17, 18]. When delivered effectively, this approach also has the potential to streamline care and ease caregivers’ burden [19, 20]. Nonetheless, to date, the number of published house call models in ALS remains limited [17, 21].

Here, we describe the ALS House Call Program (HCP) at MGH, which brings ALS expertise into patients’ home. This program reduces the burden on ALS patients of accessing care and supports patients across the disease spectrum, including in the later stages, when physical disability can isolate patients from clinic. It allows coordination between the patient’s primary ALS care team and other home care services including home aides, physical, occupational, respiratory, and speech therapists, visiting nurses, and home palliative care/hospice services. The objective of this paper is to report on the program’s impact on patient care, identify trends in service utilization, and highlight areas for growth.

## 2. Methods

### 2.1. Population

A retrospective chart review was conducted on people living with ALS and primary lateral sclerosis (PLS) who were seen at the MGH ALS multidisciplinary clinic and participated in the MGH’s ALS HCP from January 1, 2024, to December 31, 2024. Data were extracted from electronic health records using a hand review of charts and extracting information for tabulation, focusing on patient demographics, details of home visit personnel, and procedures performed during these visits.

### 2.2. Program History and Overview

**FIGURE 1:**
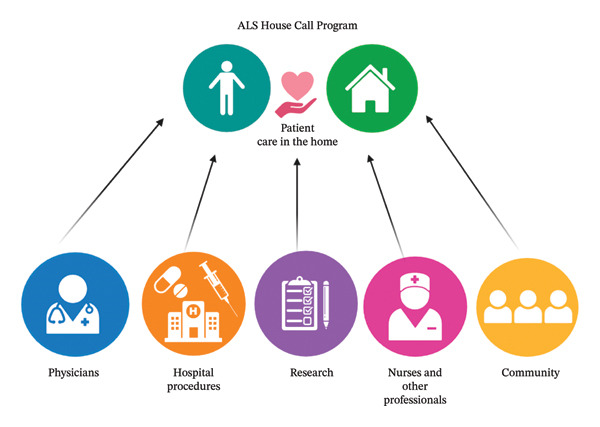
Collaboration of hospital and community teams in the amyotrophic lateral sclerosis house call program. Hospital and community healthcare providers collaborate closely to provide integrated, patient‐centered care. The interconnected components—physicians, nurse practitioners, nurses, other medical professionals, and community workers—provide care, procedures, support, and research in the home, ensuring comprehensive care coordination. Interventions usually performed in a hospital setting (such as gastrostomy tube exchanges, slow vital capacity, and maximum inspiratory pressure) can be performed in the home setting. Nurses and other professionals perform visits, while community resources address the broader needs of amyotrophic lateral sclerosis (ALS) patients and their families. Research integration allows for the incorporation of the latest ALS treatment innovations. The ALS house call program facilitates coordination among the patient’s primary care team and home care services, promoting a holistic, patient‐centered approach to ALS care. Figure 1 was created using BioRender (Scientific Image and Illustration Software). Abbreviation: ALS, amyotrophic lateral sclerosis.

**FIGURE 2:**
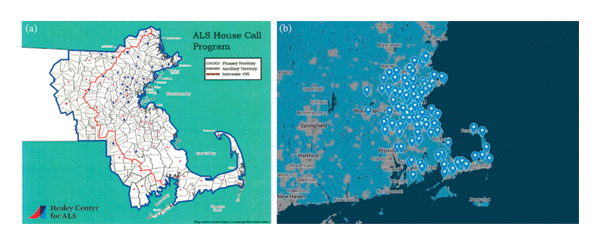
Geographic distribution of service area and patient locations. Figure (a) shows the overall geographic distribution of the program, while Figure (b) illustrates the locations where services were provided. Residential addresses were identified using mapcustomizer.com, and the base map was downloaded to visualize the spatial distribution. Abbreviation: ALS, amyotrophic lateral sclerosis.

The ALS HCP at MGH emerged from the need to deliver comprehensive care to patients followed by the MGH multidisciplinary ALS clinic who were unable to continue to travel. Since the founding of the clinic, staff sporadically performed house calls, informing the understanding that care delivered in the home both maintains the connection between the patient and the clinic and provides uniquely focused and informed care. In 2017, the program was formally established as “the Daniella Lipper ALS HCP” in honor of its generous donor. This initiative was launched as a collaboration between the ALS multidisciplinary clinic at MGH and Compassionate Care ALS (CCALS), a nonprofit organization that supports people living with ALS and their families/caregivers, with visionary philanthropic funding to support it. The Daniella Lipper ALS HCP at MGH is designed to supplement in‐clinic and telemedicine care and maintain continuity through routine scheduled visits, facilitating coordination among the patient’s primary care team and home care services (Figure 1). It operates across 8 out of 14 Massachusetts counties in Eastern Massachusetts: Worcester, Middlesex, Barnstable, Norfolk, Plymouth, Essex, Suffolk, and Bristol. A map of the serviced locations and patient distribution is shown in Figure 2.

### 2.3. Multidisciplinary Team

The Daniella Lipper ALS HCP team is currently comprised of members from MGH and CCALS. Additional details of each role and their responsibilities can be found in Table 1.

**TABLE 1:** The ALS House Call Program roles and responsibilities.

Organization	Role	Responsibilities^§^
MGH	Medical Doctor	Directs the ALS HCP
	Nurse practitioners	Help to lead the program, perform house calls including formal face‐to‐face visits for insurance purposes, provide clinical care and procedures
	Registered nurses	Perform house calls to provide clinical care
	Co‐ops from Northeastern University^†^	Handle referrals, schedule patients, maintain records, and integrate clinical and research aspects of the program

CCALS	Chief Executive Officer	Oversees the ALS HCP
	Communication Coordinator	Manages communication and coordination within the team
	Senior Family Care Liaisons	Perform house calls and provide support to families
	Social Worker	Offers additional support, particularly in end‐of‐life decisions

Abbreviations: ALS, amyotrophic lateral sclerosis; CCALS, Compassionate Care ALS; HCP, House Call Program; MGH, Massachusetts General Hospital.

^§^Nurse practitioners, registered nurses, and senior family care liaisons are all primarily involved in performing home visits within the ALS HCP. Each of these roles contributes to delivering comprehensive care and support to ALS patients and their families in their home environment.

^†^Co‐ops are students enrolled in cooperative undergraduate education programs that integrate academic study with practical work experience, allowing them to gain hands‐on skills in their field while earning academic credit.

### 2.4. House Call Model

The Daniella Lipper ALS HCP is organized in two flexible ways: (1) *routine visits* every 2 to 3 months (of note, recurring follow‐up appointments may occur while the patient is still attending in‐person clinic appointments) and (2) *as needed* or *episodic visits* to ensure timely and targeted responses to specific care priorities. While a few patients may be seen only through house calls, most patients seen in the ALS HCP are also seen intermittently in clinic and by telemedicine to provide robust clinical care and support. Neurologists, palliative care physicians, and physical and speech therapists do not routinely conduct house calls; however, they may connect with patients via video calls or attend home visits upon referral by the program team.

While more than half of the house call visits are conducted jointly by the MGH and CCALS teams, visits are also carried out exclusively by either the MGH or the CCALS teams.

The visits address a wide range of needs experienced by people with ALS. These include comprehensive medical assessments (i.e., evaluations of swallowing function, sleep patterns, and mood changes) along with management of complex symptoms, spirometry testing, G‐tube replacement, and review of home medical equipment. During the visits, a supportive environment is provided for sensitive conversations about goals of care and advance care planning, including assistance with completing Medical Orders for Life‐Sustaining Treatment (MOLST) forms [22], to ensure that care decisions align with the patient’s values and preferences. When appropriate, opportunities to participate in research studies are also discussed, including but not limited to clinical trials, observational studies, expanded access programs, registry studies, and biorepositories.

The team also assists with health insurance consultations and provides access to support groups for patients and their caregivers.

### 2.5. Key Home Procedures

#### 2.5.1. Spirometry

The Daniella Lipper ALS HCP has had the opportunity to examine multiple methodologies for measuring vital capacity, a crucial parameter in monitoring ALS disease progression [23]. At the beginning of the program, spirometry was conducted by trained healthcare providers within the patient’s home. In response to the COVID‐19 pandemic, the program adapted to facilitate home‐based, self‐administered spirometry for patients, following preliminary findings from a Pennsylvania State University (US) study suggesting that home‐based, self‐administered spirometry could enhance timely qualification for bilevel positive airway pressure (BiPAP) [24]. Through this initiative, patients used the ZEPHYRx remote respiratory monitoring remote respiratory monitoring device to monitor their slow vital capacity (SVC) on a weekly basis. The collected data were transmitted via a mobile app and displayed on a clinician‐facing dashboard, enabling continuous and remote respiratory assessment. In 2023, the program transitioned back to provider‐administered spirometry, the approach still in use.

#### 2.5.2. G‐Tube Exchange

G‐tubes, commonly used by people with ALS who develop dysphagia, can be placed either radiologically or surgically, with radiologically placed tubes typically requiring replacement every 6 to 9 months. Since 2021, the MGH HCP nursing team has performed G‐tube exchanges at home and provided chemical cautery to treat hypergranulation tissue around the G‐tube stoma, a common complication that can cause bleeding and discomfort.

### 2.6. Research

Patients from the HCP participated in a longitudinal biomarker study using cytology time of flight (CyTOF) analysis to examine immune cell function.

### 2.7. Support and Equipment

The CCALS team addresses critical aspects of ALS management, including referral to social workers and occupational therapists to assess social, personal, and home equipment needs such as solutions for essential items (i.e., wheelchairs) and adjustments for home accessibility (i.e., alternative stairs, shower, and/or bed arrangements). CCALS also offers guidance on health insurance and provides access to support groups for patients and their caregivers.

### 2.8. Financial Model

The Daniella Lipper ALS HCP has always been funded by philanthropy. In our care model, house calls can be billed by NPs using a designated house call code (Code 99350); however, this billing code does not reimburse for travel time, which represents a significant operational cost. Additionally, our program includes independent RN‐led house calls, which are not reimbursable through insurance. Consequently, philanthropic support is vital to defray shortfalls from clinical visits.

## 3. Results

### 3.1. Demographic and Clinical Visit Data

In this retrospective study, we focused on 2024 and analyzed data from 142 patients who participated in the Daniella Lipper ALS HCP at MGH. In 2024, a total of 959 visits were conducted. Notably, since the program’s inception in 2017, 3562 visits have been conducted.

In 2024, the MGH ALS multidisciplinary clinic provided care for 1132 total patients, with 671 patients residing in Massachusetts. Of these, 618 residents of the Eastern Massachusetts counties were served by the Daniella Lipper ALS HCP. In 2024, 142 (23%) out of the 618 Eastern Massachusetts residents participated in the ALS HCP. Of these 142 patients (average age: 68 years, range: 36–93; 47.9% female), 137 (96.5%) were diagnosed with ALS and 5 (3.5%) with PLS. A total of 61 (43%) out of the 142 patients were seen solely in their homes, and of these, 19 (13.4%) were also followed with telemedicine. An overview of the demographic and clinical characteristics is provided in Table 2.

**TABLE 2:** Demographic and clinical characteristics of patients in the Daniella Lipper ALS House Call Program.

	**All patients** ***N* = 142**	**ALS patients** ***N* = 137 (96.5%)**	**PLS patients** ***N* = 5 (3.5%)**

Female, No. (%)	68 (47.9%)	64 (46.7%)	4 (80%)
Age (years), average (range)	68 (36–93)	67 (36–92)	79 (66–93)
ALS onset, No. (%)			
Limb	104 (73.2%)	100 (73.0%)^†^	4 (80%)
Bulbar	32 (22.5%)	31 (22.6%)	1 (20%)
Respiratory	4 (2.8%)	4 (2.9%)	0
Torso	2 (1.4%)	2 (1.5%)	0
G‐tube, No. (%)	60 (42.2%)	60 (43.8%)	0
NIV, No. (%)	80 (56.3%)	79 (57.6%)	1 (20%)
Invasive ventilation, No. (%)	19 (13.4%)	19 (13.9)	0

Abbreviations: ALS, amyotrophic lateral sclerosis; G‐tube, gastrostomy tube; NIV, noninvasive ventilation; PLS, primary lateral sclerosis.

^†^One of the limb‐onset ALS patients presented initially with cognitive deficit (this started 2 years prior to hand weakness).

### 3.2. Visits per Week

The average number of house calls per week was 7.45, ranging from a minimum of 1 visit to a maximum of 16 visits per week. Each type of visit provided to patients cared for in the HCP is reported in Table 3.

**TABLE 3:** Total visit types for Daniella Lipper ALS house call patients in 2024.

Total number of patients	142
Total visits	959
MGH ALS HCP visits with CCALS	186
MGH ALS HCP visits without CCALS	160
MGH ALS HCP visits, with or without CCALS	346
With G‐tube exchange	44 (12.7%)^§^
With SVC	43 (12.4%)^†^
With MIP	16 (4.6%)
MGH in‐person clinic visits	223
MGH telemedicine visits	228
CCALS solo home visits	162

*Note:* The total number of visits exceeds the number of patients because individual patients may have received multiple visits and visits of multiple types during the observed period (house call visits, video televisits, and in‐clinic visits). On average, each patient received 7.45 home visits during the study period, ranging from a minimum of 1 visit to a maximum of 16 visits per week.

Abbreviations: ALS, amyotrophic lateral sclerosis; CCALS, Compassionate Care ALS; G‐tube, gastrostomy tube; HCP, House Call Program; MGH, Massachusetts General Hospital; MIP, maximum inspiratory pressure; SVC, slow vital capacity.

^§^G‐tube replacement was attempted 45 times, with one unsuccessful procedure.

^†^SVC was attempted 50 times, with 7 unsuccessful collections due to disease progression.

### 3.3. Travel

Among 346 visits in total in 2024, the HCP team collectively traveled 8610.1 miles, with an average distance of 30.32 miles (range: 1.9–117) between the patient’s home and MGH.

### 3.4. Key Home Procedures

#### 3.4.1. Spirometry

Out of the program’s 346 home visits conducted in 2024, SVC and maximum inspiratory pressure (MIP) were performed during 43 (12.4%) and 16 (4.6%) visits, respectively (Table 3), potentially preventing 59 hospital visits.

#### 3.4.2. G‐Tube Exchange

Within the 346 total home visits conducted in 2024 through the HCP, G‐tube exchanges were performed during 44 (12.7%) visits (Table 3), potentially leading to 44 avoided hospital visits.

### 3.5. Research

In 2024, at least 20 (14%) out of the 142 HCP patients participated in ALS research. Out of the 20, eight participated in expanded access protocols (EAPs), including Pridopidine EAP 1 and Pridopidine EAP 2 (NCT06069934), RAPA EAP (NCT06169176), CNMAu8 EAP (NCT06408727), and RNS60 EAP [25].

## 4. Discussion

The Daniella Lipper ALS HCP at MGH represents an innovative model of care for people living with ALS. The program is an extension of the multidisciplinary ALS clinic at MGH, delivering care by providers with ALS expertise who are familiar with the patients from their clinic visits. The program originated from sporadic house calls performed by the clinic team and subsequently grew through the generosity of patients, many of whom contributed as donors, as well as other supporters. In 2024, the program facilitated 44 G‐tube exchanges and 43 SVC assessments in the patient’s home, with the team traveling over 8000 miles. This helped minimize the travel demands placed on patients and their caregivers while ensuring quality care and disease monitoring.

The Daniella Lipper ALS HCP has also enhanced access to clinical research for patients in their homes, thereby reducing the burden associated with participation in research studies and broadening the spectrum of patients involved.

House calls contribute to patient‐centered continuous care, particularly during the advanced stages of the disease when mobility is impaired [26]. At the same time, barriers to developing an HCP for people living with ALS exist. Managing “windshield time” and efficiently visiting patients with a rare disease over a large geographic area require careful planning. Additionally, maintaining close communication between the HCP and clinic staff can be challenging due to time constraints, yet it is integral to the successful coordination of care across HCP and in‐clinic visits.

The Daniella Lipper ALS HCP has a number of strengths. It ensures continuity of care and tailored support with its staff of ALS clinicians, who know the patients and caregivers from clinic. The program brings ALS experts directly to people living with ALS, eliminating the need for travel to the clinic and provides a broad range of home‐based clinical procedures, including G‐tube exchanges and SVC assessments. Finally, the program expands clinical research access to further improve accessibility.

At the same time, the program has limitations. These findings reflect the experience of a single center with a relatively small sample size, which may limit generalizability. Philanthropic support may not be available in all settings, requiring alternative funding mechanisms. Additionally, the costs and logistical burden of staff travel time need to be considered thoughtfully.

This program marks a promising start to stronger collaboration between ALS clinics and ALS community organizations. Future program development will focus on expanding home services, such as performing tracheostomy changes in patients’ homes. Further research is needed to evaluate the clinical and financial impact of house calls throughout ALS progression, as well as their effect on hospital utilization. The development of evidence‐based home care guidelines for healthcare professionals and clinics should also be undertaken [27–30]. Finally, scalability will require sustainable funding models to support additional trained staff, ongoing professional development in ALS care, and a robust administrative and logistical infrastructure. The house call billing code 99350 can be particularly important for financial sustainability. This code allows providers to bill for prolonged outpatient services, which can be essential for programs like the ALS HCP that require extensive patient interaction and care coordination. However, this code excludes reimbursement for travel time, a major operational cost. At present, the program is partially dependent on philanthropic funding. Alternative funding mechanisms, such as institutional partnerships and grant support, may be critical. Broader implementation could be bolstered by advocating for improved reimbursement pathways for house calls in serious and physically debilitating illnesses.

**Nomenclature**
ALS: Amyotrophic lateral sclerosis
BiPAP: Bilevel positive airway pressure
CCALS: Compassionate Care ALS
CyTOF: Cytology time of flight
DO: Doctor of Osteopathy
G‐tube: Gastrostomy tube
HCP: House Call Program
MD: Medical Doctor
MGH: Massachusetts General Hospital
MIP: Maximum inspiratory pressure
MOLST: Medical Orders for Life‐Sustaining Treatment
NIV: Noninvasive ventilation
NP: Nurse practitioner
PLS: Primary lateral sclerosis
RN: Registered nurse
SVC: Slow vital capacity

## Funding

The Daniella Lipper ALS House Call program has been funded by the EGL Foundation. Since its inception, the ALS House Call program has also received funding from ALS One, Massachusetts Chapter of the ALS Association, and numerous individual donors.

## Ethics Statement

We confirm that we have read the Journal’s position on issues involved in ethical publication and affirm that this report is consistent with those guidelines.

## Conflicts of Interest

Dr. Sabrina Paganoni reports research grants from Amylyx Therapeutics, Revalesio, Eledon, Alector, UCB Pharma, Biohaven, Clene Nanomedicine, Prilenia Therapeutics, Seelos, Calico, Denali, NIH, DoD, and the Muscular Dystrophy Association and reports consulting fees from Amylyx, Arrowhead, Biogen, BMS, Clene, Cytokinetics, Eikonizo, J&J, Merck, PharmAust, Prilenia, and Sola. She has been a paid educational speaker for Medscape, PeerView, and i3Health.

Dr. James D. Berry reports research grants from Alexion, Biogen, Brainstorm Cell Therapeutics, MT Pharma of America, ProJenX, Rapa Therapeutics, UniQure, the ALS Association, the Muscular Dystrophy Association, ALS One, ALS Finding a Cure, DoD, and NINDS, reports personal consulting fees from Biogen, Marvel Biome, MT Pharma of America, Projects in Knowledge, and Roon, and has been a paid DSMB member for Sanofi.

Dr. Doreen Ho has received research funding from Prilenia, VectorY, RA Pharma, Biohaven, Clene, Seelos, Calico, Denali, Biogen, Transposon, Sanofi, the Neurodegenerative Alzheimer’s Disease and Amyotrophic Lateral Sclerosis Basket Trial, and Genentech. Dr. Ho has received compensation for serving on Sanofi, Alexion, Scholar Rock, and Biogen Advisory Boards.

Dr. Merit E. Cudkowicz reports consulting/financial relationships with Neurosense, Biogen, Regeneron, Transposon, Locust Walk, Arrowhead, Praxis, VectorY, Servier, Eledon, Cytokinetics Pasithea, Ono, Novartis, Roche, Denali, Immunity Pharm, RRD, Takeda, MTPC, inFlectis, QurAlis, Otsuka, Aclipse, Coya, AC Immune, and Pontifex.

The remaining authors declare no conflicts of interest.

## Data Availability

The data that support the findings of this study are available on request from the corresponding author.
